# Pharmacokinetics of Active Components From Guhong Injection in Normal and Pathological Rat Models of Cerebral Ischemia: A Comparative Study

**DOI:** 10.3389/fphar.2018.00493

**Published:** 2018-05-15

**Authors:** Li Yu, Hao-fang Wan, Chang Li, Jie-hong Yang, Hui-fen Zhou, Hai-tong Wan, Yu He

**Affiliations:** ^1^College of Life Science, Zhejiang Chinese Medical University, Hangzhou, China; ^2^College of Pharmaceutical Science, Zhejiang Chinese Medical University, Hangzhou, China; ^3^College of Basic Medical Sciences, Zhejiang Chinese Medical University, Hangzhou, China

**Keywords:** Guhong injection, aceglutamide, HSYA, cerebral ischemia, pharmacokinetics, comparison

## Abstract

**Background and Objectives:** Guhong Injection (GHI) is usually administered for the treatment of stroke in clinics. Aceglutamide and hydroxyl safflower yellow A (HSYA) are its key ingredients for brain protective effect. To investigate the pharmacokinetics of aceglutamide and HSYA under pathological and normal conditions, the pharmacokinetic parameters and characteristics of middle cerebral artery occlusion (MCAO) and normal rats given the same dosage of GHI were studied compared.

**Methods:** 12 SD rats were divided into two groups, namely, MCAO and normal groups. Both groups were treated with GHI in the same dosage. Plasma samples were collected from the jaw vein at different time points and subsequently tested by high-performance liquid chromatography (HPLC).

**Results:** After administration of GHI, both aceglutamide and HSYA were immediately detected in the plasma. Ninety percent of aceglutamide and HSYA was eliminated within 3 h. For aceglutamide, statistically significant differences in the parameters including AUC_(0−t)_, AUC_(0−∞)_, AUMC_(0−t)_, AUMC_(0−∞)_, C_max_ (*P* < 0.01), and V_z_ (*P* < 0.05). Meanwhile, compared with the MCAO group, in the normal group, the values of AUC_(0−t)_, AUMC_(0−t)_, VRT_(0−t)_, and C_max_ (*P* < 0.01) for HSYA were significantly higher, whereas the value of MRT_(0−t)_ was significantly lower in the normal group.

**Conclusions:** The *in vivo* trials based on the different models showed that, the pharmacokinetic behaviors and parameters of aceglutamide and HSYA in GHI were completely different. These results suggest that the pathological damage of ischemia-reperfusion has a significant impact on the pharmacokinetic traits of aceglutamide and HSYA.

## Introduction

Stroke is defined as an acute neurologic dysfunction of vascular origin with sudden or rapid occurrence in response to symptoms and signs of involvement of the focal area in the brain (Listed, 1989). There are two main types of strokes, and approximately 80% are acute ischemic strokes (Li et al., 2012). Unfortunately, despite the advanced therapeutic methods and techniques available at present, stroke has remained a grave problem worldwide. Stroke is the leading cause of death in China and is fifth in the United States (Howard et al., 2017; Otite et al., 2017). Thrombolytic recombinant tissue plasminogen activator (rt-PA), the only FDA-approved medication for curing stroke, is often limited by a narrow time window and the potential risk of hemorrhagic transformation (Young et al., 2007; Zhang et al., 2012). Therefore, development of effective medicine for the treatment of cerebral ischemia is imperative. Modern medicine has changed from a single drug model to a multi target (combined drug) model. Drug combination therapy has gradually become a trend in the conception and development of new drugs. Guhong injection (GHI) is an effective traditional Chinese and Western pharmaceutical preparation clinically applied for cerebrovascular diseases such as cerebral embolism, hemorrhage, and mental deterioration (Zhao et al., 2006).

**Figure 1:**
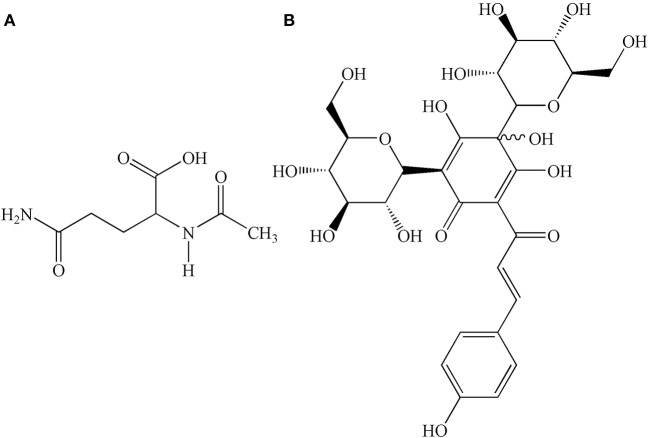
Chemical structures of **(A)** aceglutamide, molecular formula: C_7_H_12_N_2_O_4_; **(B)** HSYA, molecular formula: C_27_H_32_O_16_.

GHI, a novel compound preparation, is composed of aceglutamide and aqueous extract of safflower (*Carthamus tinctorius* L.). The combination of these two drugs has a synergistic effect on blood coagulation, causing anti-platelet aggregation, dilatation of blood vessels, and anti-inflammatory effects (Ai et al., 2017). Aceglutamide, whose structure is shown in Figure 1A, is a glutamine acetyl derivative, which is a liquid-stable source of glutamine. Aceglutamide has the same pharmacological effect (Zhang et al., 2015) as that of glutamine, which could significantly improve the clinical prognosis of patients who had a stroke (Wasa et al., 2005). Owing to its the effect of promoting blood circulation and relieving pain, safflower is clinically used for the treatment of cardiovascular and cerebrovascular diseases such as myocardial ischemia, cerebral thrombosis, and coronary heart disease (Xia et al., 2013). Hydroxyl safflower yellow A (HSYA), whose chemical structure is shown in Figure 1B, is one of the most effective water-soluble components in safflower. The protective effect of HSYA in ischemic stroke including anti-inflammation, anti-oxidation, anti-calcium dysregulation, anti-thrombosis, and anti-coagulation, had been reported in previous studies (Hunt et al., 1978; Sun et al., 2010; Fan et al., 2014).

Researchers have studied the effects of some ingredients such as HSYA on the pharmacokinetics and pharmacodynamics of GHI (Shi et al., 2015). However, to our knowledge, no study has evaluated the pharmacokinetic difference between normal and MCAO rats after injecting GHI. The aim of this study was to investigate the pharmacokinetics of aceglutamide and HSYA in MCAO/normal rat models and decipher a possible link between the absorption of GHI, especially the evaluated contents, and pharmacokinetic parameters of aceglutamide and HSYA in normal and MCAO rats. The corresponding validated method was applied to control the quality of aceglutamide and HSYA and supplied additional information in the clinical usage of Chinese drugs.

## Materials and methods

### Chemicals and reagents

GHI was obtained from Buchang Pharmaceutical Inc. (Shanxi, China). And we previously studied the preparation of GHI (Ai et al., 2017) and established the fingerprint of GHI (He et al., 2015b; Ai et al., 2017). According to the corresponding quality control standard, the content of aceglutamide should be in the range of 27–33 mg/mL and the content of HSYA should be in the range of 0.410–437 mg/mL when detected by HPLC. Aceglutamide, HSYA and riboflavin standards were purchased from Yuanye Biotechnology Co., Ltd (Shanghai, China) to determine concentrations in plasma. Acetonitrile and methanol were of high HPLC grade (Merck, Germany). Trifluoroacetic-acid was obtained from Chengdu Kelon chemical reagents Co., Ltd. (Sichuan, China). Pure water was supplied by a Millipore pure water system (Millipore, America).

### Focal middle cerebral artery occlusion

Male Sprague–Dawley (SD) rats (260–300 g, Certification No.SCXK 2014-0001) were obtained from the animal experiment center of Zhejiang Chinese Medical University, and fasted for 12 h with free access to water before surgery. Animal welfare and experimental procedures were strictly in accordance with the Regulation for the Administration of Affairs Concerning Experimental Animals (State Science and Technology Commission, 1988) and approved by the Animal Subjects Review Board of Zhejiang Chinese Medical University.

Rats were anesthetized with 10% chloral hydrate solution, afflicted with stroke using a middle cerebral artery occlusion (MCAO) operation according to the method of Longa et al. (1989), with minor modification. Following a midline incision in the neck of each rat, the right common carotid artery (CCA), the external carotid artery (ECA), and the internal carotid artery (ICA) were separated. After the ECA and the CCA were ligated, a 0.28 mm polylysine-coated nylon monofilament was inserted into the MCA through ICA to maintain a state of ischemia for 1 h. The monofilament was slowly withdrawn, and the animals were returned to their cages. After 23 h of reperfusion, the operated rats were scored and those manifesting stroke were chosen. During the surgery, the core temperature was maintained at 37 ± 0.5°C by a thermostatically controlled heating pad.

After reperfusion for 23 h following 1 h of cerebral ischemia, the operated rats were observed and graded (Bederson et al., 1986). Rats with contralateral hemiparesis and Horner's syndrome (1 point), contralateral orbiting (2 points), contralateral tumble (3 points), and paralysis without consciousness (4 points) were chosen as stroke rats.

### Instrumentation and chromatographic conditions

The analysis was performed on a Waters e2695 series system (Waters technologies, USA) equipped with a 2489 UV-vis detector. Chromatographic separation was achieved on an Alltima C_18_ (4.6 × 250 mm, 5.0 μm) analytical column at 30°C. A gradient elution program was conducted for chromatographic separation with mobile phase trifluoroacetic acid aqueous solution (A, pH 3.5) and acetonitrile (B) as follows: 0–15 min (A: 98–85%), 15–30 min (A: 85%). The flow rate was 1.0 mL/min. Ten microliters of sample was injected automatically. The detector operated at 210 nm for detecting aceglutamide and 403 nm for detecting HSYA.

### Sample preparation

The thawed plasma samples (100 μL) were pipetted into centrifuge tubes, mixed with 300 μL methanol and 100 μL riboflavin standard (0.1 mg/mL). The mixture was vortexed for 30 s and centrifuged at 12,000 rpm for 10 min. Supernatant was removed and evaporated under N_2_ stream in a water bath at 60°C. The residue was dissolved by the mobile phase and filtered. Finally, 10 μL of supernatant was injected into the HPLC system for analysis.

### Method validation

The reference standards of aceglutamide and HSYA were accurately weighed and dissolved in methanol. Their final concentrations were both 1.0 mg/mL, this was called the standard stock solution. By spiking 100 μL blank serum with appropriate volumes of the standard stock solution, the aceglutamide (10, 25, 50, 250, 500, and 1,000 μg/mL) and HSYA (0.33, 1, 2, 5, 10, and 40 μg/mL) reference standard solutions at six different concentrations were prepared. These standard solutions were subjected to the entire analytical procedure and used to validate the linearity, precision, accuracy, recovery, and stability (quality control, QC) of samples in this method.

### *In vivo* pharmacokinetic study

Rats were divided into two groups (*n* = 6): MCAO and normal groups. After reperfusion for 23 h, rats in MCAO group were injected with GHI (2.10 mL/kg, which was calculated according to the clinical human dose) in the tail vein. Meanwhile, rats in the normal group were administered the same dose of GHI. After intravenous injection, 0.5 mL plasma samples were collected into heparinized tubes from the jaw vein at the time points of 2, 5, 10, 15, 20, 30, 45, 60, 90, 120, 180, 240, and 360 min, respectively. After centrifugation at 4,000 rpm for 10 min, plasma samples were stored at −20°C and analyzed within 1 week.

### Statistical analysis

The pharmacokinetic parameters were calculated using a non-compartment model in the software Kinetica version 4.4 (Innaphase, MA, USA). Statistical comparisons among groups were performed with SPSS Version 17.0 (SPSS Inc., Chicago, IL, USA) using the analysis of variance (ANOVA). All the data were expressed as the mean ± standard deviation (SD).

## Results

### Selectivity

**Figure 2:**
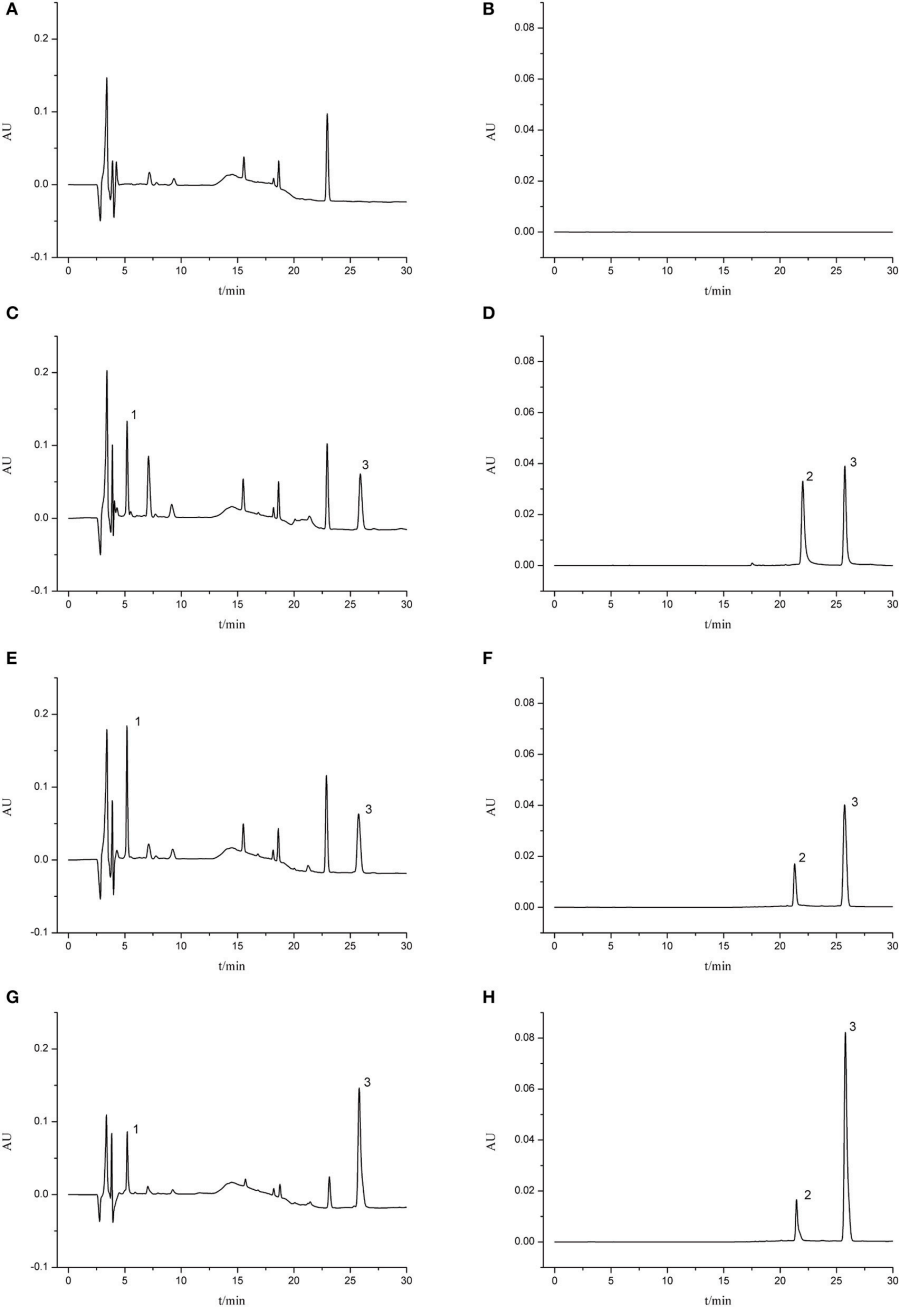
Typical chromatograms of rat plasma. **(A)** Blank serum sample (210 nm); **(B)** blank serum sample (403 nm); **(C)** blank serum sample spiked with aceglutamide and riboflavin (210 nm); **(D)** blank serum sample spiked with HSYA and riboflavin (403 nm); **(E)** MCAO group 5 min after GHI injection (210 nm); **(F)** MCAO group 5 min after GHI injection (403 nm); **(G)** Normal group 5 min after GHI injection (210 nm); **(H)** Normal group 5 min after GHI injection (403 nm) (1: aceglutamide; 2: HSYA; 3: riboflavin).

The validated HPLC method was successfully applied to the pharmacokinetic study of aceglutamide and HSYA after injection of GHI. Typical chromatograms of different samples are presented in Figure 2. No significant endogenous analyte was observed near the retention times of aceglutamide (5.18 min), HSYA (21.46 min), and riboflavin (25.80 min).

### Linearity and lower limit of detection

The calibration curve for aceglutamide was Y = 0.0018X+0.2656 with *r*^2^ = 0.9986 in the range of 10.0–1000.0 μg/mL. The calibration curve for HSYA was Y = 0.0242X-0.0238 with *r*^2^ = 0.9939 in the range of 0.33–40.00 μg/mL. When signal noise ratio was 3, the lower limit of detection (LLOD) for aceglutamide and HSYA were 2.50 μg/mL and 0.12 μg/mL, respectively.

### Accuracy, precision, and recovery

The intraday and interday accuracy and precision were determined by replicate analyses of samples continuously on the same day (intraday) and over 5 days (interday), respectively. These are shown in Table 1. The precisions of aceglutamide and HSYA calculated as the relative standard deviation (RSD) at various concentrations were lower than 10% for intraday and interday assays. Moreover, the accuracy of aceglutamide and HSYA calculated as the relative error (RE) at various concentrations were within 10% for samples. The results demonstrated that the accuracy and precision of this method were acceptable.

**Table 1:** Precisions and recoveries of each reference substance ($\bar{x}\pm$ *s, n* = 5).

**Analyte**	**Concentration (μg/mL)**	**Recovery (%)**	**Intraday**		**Interday**	
			**RSD (%)**	**RE (%)**	**RSD (%)**	**RE (%)**
Aceglutamide	100	96.433 ± 4.270	1.028	4.453	4.918	7.724
	250	103.538 ± 5.653	4.444	0.927	5.187	1.687
	500	93.438 ± 0.832	1.319	2.053	3.066	1.824
HSYA	4	98.189 ± 3.753	1.657	3.867	6.147	6.542
	10	101.563 ± 3.832	7.246	8.173	5.491	8.088
	20	95.624 ± 1.226	0.473	3.007	5.118	3.335

The recovery of aceglutamide and HSYA from plasma is shown in Table 1.

### Stability

Ten samples of medium- concentration mixed control plasma samples were prepared under identical conditions. Five samples were measured immediately according to the existing chromatographic conditions. The peak area of chromatograph was recorded and the true initial concentrations of aceglutamide and HSYA in plasma samples were calculated. These five samples were frozen at −20°C and thawed three times, then pretreated and injected into the HPLC. The results showed that the average concentrations of aceglutamide and HSYA in the plasma samples were 95.43 and 97.21%, respectively. The other five samples were placed at −80°C for 1 week, and assayed after thawing at room temperature. The relative standard deviations (RSDs) of aceglutamide and HSYA were 5.63 and 5.55% respectively, which demonstrated that these analytes were stable in rat plasma.

### Pharmacokinetics study

**Figure 3:**
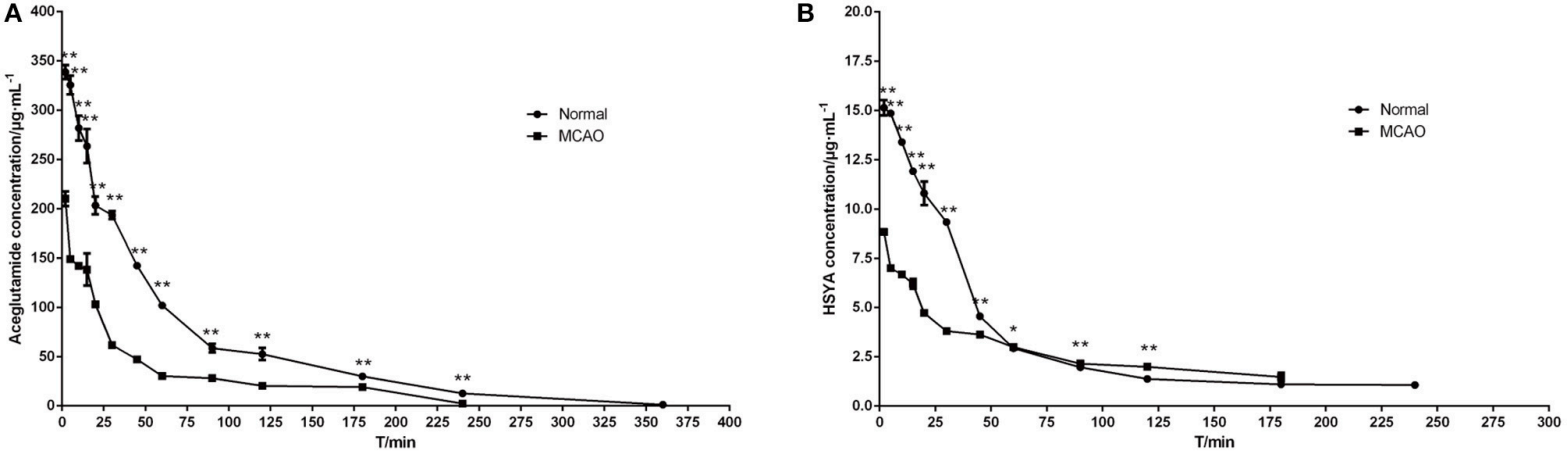
Mean plasma concentration–time curve profiles of aceglutamide and HSYA. **(A)** aceglutamide **(B)** HSYA. Values are mean ± SD of 6 rats. ^*^P < 0.05, ^**^P < 0.01, compared with MCAO group.

The mean plasma concentration-time curve profiles are illustrated in Figure 3, showing rapid absorption and excretion of aceglutamide and HSYA in all groups. Both active components could be detected in the plasma immediately after injecting GHI. After that 90% of the aceglutamide and HSYA were eliminated within 3 h. The decreasing amplitude of concentrations of aceglutamide and HSYA in MCAO rats was more significant than in the normal group. Statistically significant differences were noted among the above results.

The pharmacokinetics parameters are shown in Table 2. After injecting GHI, aceglutamide had statistically significant differences in the parameters including the AUC_(0−t)_, AUC_(0−∞)_, AUMC_(0−t)_, AUMC_(0−∞)_, C_max_ (all *P* < 0.01), and V_z_ (*P* < 0.05) between the MCAO and normal rats. At the same time, compared with the MCAO group, the values of AUC_(0−t)_, AUMC_(0−t)_, MRT_(0−t)_, VRT_(0−t)_, and C_max_ (*P* < 0.01) about HSYA were significantly higher in the normal group. The results suggest that cerebral ischemia–reperfusion reduces the absorption of aceglutamide and HSYA in GHI and influences their pharmacokinetics characteristic, especially aceglutamide. In other words, pharmacologically, GHI had a therapeutic effect on cerebral ischemia.

**Table 2:** Pharmacokinetic parameters of aceglutamide and HSYA in different groups ($\bar{x}\pm$*s, n* = 6).

**Parameters**	**Aceglutamide**		**HSYA**	
	**Normal**	**MCAO**	**Normal**	**MCAO**
AUC_(0−t)_ (μg/L·min)	21373.54 ± 548.58^ΔΔ^	7250.72 ± 3553.22	787.48 ± 7.45^**^	528.14 ± 6.00
AUC_(0−∞)_ (μg/L·min)	21540.20 ± 491.25^ΔΔ^	7373.93 ± 3613.60	952.59 ± 57.00	909.84 ± 221.11
AUMC_(0−t)_ (min·min·μg/L)	21540.20 ± 65315.79^ΔΔ^	453194.24 ± 222086.85	46493.49 ± 835.78^**^	34145.13 ± 1126.30
AUMC_(0−∞)_ (min·min·μg/L)	1542607.08 ± 69738.36^ΔΔ^	490145.45 ± 240274.57	121163.39 ± 34845.31	216471.20 ± 135541.54
MRT_(0−t)_ (min)	68.68 ± 1.40	52.09 ± 25.52	59.05 ± 1.36^**^	64.64 ± 1.49
MRT_(0−∞)_ (min)	71.61 ± 2.59	55.39 ± 27.15	125.81 ± 28.01	218.51 ± 95.87
VRT_(0−t)_ (min^2^)	4553.05 ± 205.83	3134.66 ± 1536.62	4016.79 ± 133.84^**^	2762.92 ± 126.31
VRT_(0−∞)_ (min^2^)	5668.82 ± 1238.12	3903.65 ± 1919.26	32530.29 ± 16624.99	68705.29 ± 53066.20
t_1/2z_ (min)	46.91 ± 15.87	34.48 ± 16.96	137.47 ± 32.91	169.76 ± 77.50
T_max_ (min)	2.50 ± 1.22	1.67 ± 0.82	2.00 ± 0.00	2.00 ± 0.00
V_z_ (L/kg)	9.44 ± 3.28^Δ^	16.87 ± 0.83	619.44 ± 108.31	770.99 ± 181.39
CL_z_ (L/min/kg)	0.14 ± 0.00	0.28 ± 0.14	3.16 ± 0.18	3.47 ± 0.84
C_max_ (μg/L)	338.83 ± 7.01^ΔΔ^	175.13 ± 86.11	15.15 ± 0.39^**^	8.84 ± 0.05

*Values are given as means ± SD of 6 rats*.

Δ *P < 0.05*,

ΔΔ *P < 0.01, compared with MCAO group*;

^*^P < 0.05,

** *P < 0.01, compared with MCAO group*.

## Discussion

GHI is mainly used for the treatment of cardiovascular and cerebrovascular diseases, particularly cerebrovascular diseases, such as cerebral insufficiency, cerebral thrombosis, and cerebral embolism. Our group also found that GHI has a therapeutic effect on cerebral ischemia–reperfusion injury (Dai et al., 2014; Shu et al., 2014; Ai et al., 2017; Du et al., 2017). GHI consists of aceglutamide and safflower extract. Compositional analysis showed that the content of aceglutamide and HSYA in GHI were relatively high (He et al., 2015b). Aceglutamide, which has the ability to improve the metabolism of nerve cells (Li et al., 2017), maintain the function of nerve stress and reduce the effect of blood ammonia, was selected as the first detection index in our study. Many studies have shown that HSYA is a major pharmacological component of safflower (Li et al., 2013; He et al., 2015a). With the inhibition of platelet aggregation, anti-oxidation, anti-inflammatory and other pharmacological activities, HSYA exhibits neuroprotective effect both *in vivo* (Chen et al., 2016; Du et al., 2017) and *in vitro* (Pei et al., 2017; Sun et al., 2018). Accordingly, HSYA was selected as the second test indicator. Studies on the pharmacokinetics of HSYA have been widely reported (Tian et al., 2010; Li et al., 2015). Nevertheless, to our knowledge, no pharmacokinetic study of HSYA combined with aceglutamide has been reported. Therefore, these two components were chosen to show the metabolic regularity of GHI.

The pharmacokinetics of aceglutamide and HSYA in GHI was studied under cerebral ischemia. In the early stages of the present study, we relied on our previously published procedures (Fang et al., 2018). We used this previous study to establish pharmacokinetic testing methods and to primarily grasp the pharmacokinetic characteristics of GHI under pathological conditions. Our present comparative study of GHI pharmacokinetics was mostly in the aspects of effective composition and compound administration based on pathological or normal experimental animals. However, since the research should be systematic, complete and detailed, we supplemented and perfected our previous methods. The normal rat was included to reveal the pharmacokinetic characteristics of aceglutamide and HSYA in GHI under both normal and pathological conditions. This comparative pharmacokinetics study also provided the basis for speculating on active compounds, explaining compatibility rules, and determining the mechanism of action. What's more, the study of GHI pharmacokinetics based on normal and pathological models, especially on different effects on the process of GHI absorption, distribution, metabolism, and excretion, could provide further credible guidance information for clinical medication and it be of practical significance.

In the pre-experiment of the current study, three organic reagents (methanol, acetonitrile, ethyl acetate) were adopted to remove protein macromolecules. Meanwhile, different mobile phases, such as trifluoroacetic acid aqueous solution (A, pH 3.5) and acetonitrile (B), trifluoroacetic acid aqueous solution (A, pH 3.5) and methanol (B), phosphoric acid aqueous solution (A, pH 3) and acetonitrile (B), and phosphoric acid aqueous solution (A, pH 3) and methanol (B), were tested for the isolation of aceglutamide, HSYA, and plasma interference peaks in different gradient elution times. Eventually, various outcomes showed the loss of the measured constituents and the deproteinized effect of methanol was better than acetonitrile and ethyl acetate. The mobile phase, trifluoroacetic acid aqueous solution (A, pH 3.5) and acetonitrile (B)—0–15 min (A: 98–85%), 15–30 min (A: 85%)—could maximally separate the measured constituents from the plasma interferon and the shape of the chromatographic peak was symmetric. The results of the methodological investigation confirmed that acetonitrile, and trifluoroacetic acid did not interfere with the content determination. By using the present methods, pharmacokinetics of aceglutamide and HSYA in GHI was studied swiftly and accurately.

Ischemic stroke, which covers almost all the corresponding symptoms, was the best disease model to study the pharmacology and drug metabolism of GHI. We selected the MCAO model, a well-recognized and easily replicated model, to simulate ischemic stroke. After ischemia of the middle cerebral artery, a series of cascade reactions occurred, including intracellular calcium overload, excessive release of excitatory amino acids, increased production of oxygen free radicals and destruction of blood brain barrier (BBB) (Byrne, 2012). Up to now, most reported pharmacokinetic studies of GHI (Shi et al., 2015; Chen et al., 2016) were performed on pathological animals, while the metabolic behavior of GHI might be quite different in the normal model. Hence, it was valuable to study the difference of pharmacokinetic characteristic in normal and pathological animals, which will be helpful to uncover the pharmacological mechanism of GHI and further provide guiding information for accurate and rational clinical usage of this drug.

As shown in Figure 3, the plasma concentrations of aceglutamide and HSYA in the normal group were very different from the MCAO group at 2 min (the first time point) after drug administration. This was because on the one hand, according to the corresponding quality control standard of our group, the content of aceglutamide was in the range of 27–33 mg/mL and the content of HYSA was in the range of 0.410–437 mg/mL when detected by HPLC (He et al., 2015b; Ai et al., 2017); on the other hand, HSYA has a high plasma protein binding rate. Thus, in the process of drug metabolism and the preprocessing of plasma samples, the loss rate of HSYA is high (Wang et al., 2011). Moreover, compared to HYSA, aceglutamide has better water solubility and lower protein binding rate and protein loss. Further, after 1 h ischemia of the middle cerebral artery occlusion and 23 h of reperfusion injury, the series of cascade reactions becomes increasingly. Once the drug administered, HSYA pass through the BBB to reach the targeted areas and is oxidized. Compared with MCAO group, in the normal group, at almost all time points, the blood concentrations of both aceglutamide and HSYA were significantly different. At the same time, the results of pharmacokinetic parameters showed that AUC_(0−t)_, AUMC_(0−t)_, and C_max_ of two active components were significantly higher in normal rats (Table 2). It was indicated that aceglutamide and HSYA had high absorption rates and high bioavailability in the normal group. Because of the damage to BBB, a barrier with a selective function between the brain and blood, aceglutamide, and HSYA could pass through it and reach the brain. Hence, the plasma concentrations of aceglutamide and HSYA in the MCAO group were lower than that in the normal group. That is, the values of AUC_(0−t)_, AUC_(0−∞)_, AUMC_(0−t)_, AUMC_(0−∞)_, VRT_(0−t)_, and C_max_ in the normal group were much higher than those in the MCAO group. The MRT of HSYA in the normal group was significantly higher than that in the MCAO group. It was speculated that other components in GHI, such as aceglutamide or other components in safflower, could have play a role in promoting the efficacy of HSYA.

## Conclusion

Under pathological and normal states, aceglutamide and HSYA of GHI showed distinctly different patterns in absorption and metabolism. Comparative pharmacokinetic parameters of aceglutamide and HSYA studied using normal and MCAO rat models indicated that aceglutamide was a more effective ingredient to treat brain diseases. The pathological state of MCAO could change the metabolic behavior of aceglutamide and HSYA in GHI. Our investigation of the pharmacokinetics of aceglutamide and HSYA in GHI should help to guide the clinical application of GHI and prescriptions containing aceglutamide and HSYA in patients who had a stroke. Our data will also ensure the effective clinical use of GHI, traditional Chinese medicine.

## Author contributions

LY, HaoW, JY, HaiW, and YH: Participated in research design; LY, HaoW, and HZ: Performed the experiments and data analysis; LY and CL: Contributed to the writing of the manuscript.

### Conflict of interest statement

The authors declare that the research was conducted in the absence of any commercial or financial relationships that could be construed as a potential conflict of interest.
